# Preoperative Omega-6/Omega-3 Fatty Acid Ratio Could Predict Postoperative Outcomes in Patients with Surgically Resected Non-Small-Cell Lung Cancer

**DOI:** 10.3390/curroncol29100556

**Published:** 2022-09-28

**Authors:** Carlos Déniz, Carla Raba-Parodi, Eva García-Raimundo, Iván Macía, Francisco Rivas, Anna Ureña, Anna Muñoz, Camilo Moreno, Ines Serratosa, Cristina Masuet-Aumatell, Ignacio Escobar, Ricard Ramos

**Affiliations:** 1 Thoracic Surgery Department, Hospital Universitari de Bellvitge, Carrer de la Feixa Llarga, s/n, L’Hospitalet de Llobregat, 08907 Barcelona, Spain; 2 Preventive Medicine and Public Health Department, Hospital Universitari de Bellvitge, Carrer de la Feixa Llarga, s/n, L’Hospitalet de Llobregat, 08907 Barcelona, Spain; 3 Endocrinology and Nutrition Department, Hospital Universitari de Bellvitge, Carrer de la Feixa Llarga, s/n, L’Hospitalet de Llobregat, 08907 Barcelona, Spain

**Keywords:** fatty acids, ratio omega 6/3, lung cancer, thoracic surgery, prolonged air leak

## Abstract

*Introduction:* The aim of this study was to determine whether preoperative nutritional status and inflammatory status, specifically polyunsaturated acids and the omega 6/3 ratio, would affect postoperative outcomes and complications in patients with lung cancer undergoing lung resection. *Methods:* This prospective observational study included 68 patients with early-stage non-small-cell lung cancer who were candidates for radical surgery. A complete nutritional assessment was performed. The primary study variable was postoperative complications and mortality in the first 30 days. Descriptive, bivariate, and logistic regression analyses were carried out. *Results:* A total of 50 men (73.53%) and 18 women (26.47%) underwent surgery, with a median age of 64.2 (±9.74) years. The mean omega 6/3 ratio was 17.39 (±9.45). A complication occurred in 39.7% of the study sample (*n* = 27), the most common being persistent air leak in 23.53% (*n* = 16). After performing the bivariate analysis, the only variable that remained significant was the omega 6/3 ratio; we observed that it had a prognostic value for persistent air leak (*p* = 0.001) independent of age, sex, comorbidity, preoperative respiratory function, and approach or type of surgery. The remaining nutritional and inflammatory markers did not have a statistically significant association (*p* > 0.05) with postoperative complications. However, this significance was not maintained in the multivariate analysis by a small margin (*p* = 0.052; 95% CI: 0.77–1.41). *Conclusions:* Omega 6/3 ratio may be a prognostic factor for air leak, independent of the patient’s clinical and pathological characteristics.

## 1. Introduction

Despite recent advances in early diagnosis and the potential implementation of screening programs in the near future [1], lung cancer remains the second most commonly diagnosed tumor worldwide and the leading cause of oncological deaths [2,3], with an overall 5-year survival of 10.6% [4,5].

Nutritional status has been postulated as a determining factor in the postoperative outcomes of oncology patients undergoing pulmonary resection [6]. A good nutritional status helps ensure a good immunological response; it is, therefore, pertinent to carry out a complete nutritional assessment that includes, among other variables, the status of micronutrients such as omega fatty acids. Omega-3 fatty acids are polyunsaturated fatty acids characterized in their chemical structure by the presence of a double bond three atoms away from the methyl terminal [7].

The modern diet (influenced by Anglo-Saxon countries) has a great imbalance in this aspect, as it contains a high proportion of lipids, with an omega-6 (ω-*6*) polyunsaturated fatty acid content much higher than the omega 3 (ω-*3*) content. It is suggested to maintain the ratio of omega-6/omega-3 consumption in the range from 1/5 to 1/10 and, ideally, as in Japanese society, from 1/2 to 1/4. These fatty acids are reported as having proinflammatory and anti-inflammatory functions, respectively [7,8]. The right balance between omega-6 and omega-3 is essential for the correct function of cell membranes, enzyme activity, and genetic expression.

Previous studies on this subject in patients with lung cancer have mainly been conducted in patients with advanced-stage disease, and omega-3 fatty acid supplementation has been reported to have positive effects on quality of life and physical activity during multimodal treatments [9,10].

We, therefore, sought to study the preoperative omega 6/3 ratio, among other nutritional and inflammatory variables, and their effects on postoperative outcomes in patients with early-stage lung cancer.

## 2. Material and Methods

### 2.1. Study Design

This was a prospective observational study (case series) on preoperative nutritional status in patients with early-stage non-small-cell lung cancer who were candidates for radical surgery. The study was carried out in accordance with good clinical practice and was approved by the hospital’s ethics committee. All participants gave signed informed consent to participate in the study.

#### 2.1.1. Inclusion Criteria

1. Adult patients (aged 18 years or older), male or female, of any race/ethnicity;
2. Patients who gave informed consent to participate;
3. Patients with early-stage non-small-cell lung cancer who were candidates for surgical treatment, diagnosed before or during surgery.

#### 2.1.2. Exclusion Criteria

1. Patients who did not consent to participate in any of the study phases;
2. Aged younger than 18 years;
3. Locally advanced lung cancer on neoadjuvant treatment;
4. History of rheumatologic, systemic or hepatic disease, or immunodeficiency;
5. Infection prior to surgery requiring treatment with a specific antibiotic;
6. Patients who declined surgery.

#### 2.1.3. Sample Size

It was intended to recruit 100 patients to make a model of logistic regression for the main analysis. Following the conservative rule of 10 events per independent variable and taking into account between 10 and 15% of patients with some postsurgical complication in the first 30 days, it would be a sufficient sample size to estimate the model. Data collection started in September 2017 and ceased in 2020 due to the COVID-19 situation, with a total of 71 patients recruited.

### 2.2. Data Collection and Analysis

Data were collected on demographics, past medical history, respiratory function tests, American Society of Anesthesiologists (ASA) classification, Charlson Index, tumor characteristics, type of surgery, and surgical approach. A preoperative nutritional assessment and preoperative blood tests, including nutritional and inflammatory markers, were performed.

The postoperative variables analyzed were drain duration, persistent (>5 days) air leak, drain output, and hospital length of stay. Also included were complications, mortality, and grade of complication according to the Clavien–Dindo classification (10).

On the day of surgery, a blood sample was taken to analyze the inflammatory and nutritional parameters and the proportion of omega fatty acids to calculate the omega 6/3 ratio.

#### 2.2.1. Assessment of Inflammatory Status

The neutrophil/lymphocyte ratio (NLR), platelet/lymphocyte ratio (PLR), lymphocyte/monocyte ratio (LMR), and systemic immune-inflammation index (SII) were calculated. C-reactive protein (CRP), transferrin, and other inflammatory variables were measured.

#### 2.2.2. Nutritional Assessment

All the patients were seen between 15 and 30 days before surgery by a dietician who performed a preoperative nutritional assessment that included the following: weight, height, bioelectrical impedance analysis, dynamometry, overall subjective assessment (OSA), body mass index (BMI), nutritional risk index (NRI), nutritional prognostic index (NPI), assessment of adherence to the Mediterranean diet, assessment of malnutrition status using the Chang method, International Physical Activity Questionnaire (IPAQ), fat-free mass (FFM), body cell mass index (BCMI), fat mass (FM), and malnutrition screening tool (MST).

#### 2.2.3. Quantification of Blood Omega 6/3 Ratio

A 5 mL heparinized blood sample was taken after anesthetic induction and before starting surgery after an overnight fast. It was allowed to clot for at least 30 min, then centrifuged (10 min) at 700× *g* within 1 h of being taken. The samples were aliquoted and kept frozen at −70 to −80 °C until analyzed (maximum 1 year). Quantification was by gas chromatography and spectrometry performed by the Scientific Department of the University of Barcelona.

### 2.3. Statistical Analysis

All collected data were stored in a Microsoft Access database, with the coding of patients who were assigned a number at study inclusion (pseudonymization).

All the patient variables included were described for the whole sample. We used descriptive statistics according to the nature of the variable. Continuous variables were described using number of valid observations with measures of central tendency (mean or median) and dispersion (standard deviation, interquartile range) and certain other descriptors of interest (minimum, first quartile, third quartile, maximum). Categorical variables were described as the number of valid observations and percentage.

A cutoff was calculated for the variable omega 6/3 ratio using the Youden index, and its sensitivity and specificity were studied using the area under the curve (AUC).

The Kolmogorov–Smirnov test was used to determine normality of distribution of the quantitative variables. In the bivariate analysis, we used the Student’s *t*-test for quantitative variables that followed a normal distribution and the Mann–Whitney U test for those that did not. For qualitative variables, the Chi-squared test was used.

For the multivariate analysis, logistic regression was performed. Potential confounding factors considered for the multivariate model were age, sex, preoperative BMI, preoperative BCMI, history of COPD, drain duration, persistent air leak, and NPI.

The conditions of application of the models were validated, and the 95% confidence intervals (95% CI) of the estimator were calculated. Statistical significance was set at the <0.05 confidence level. The statistical package SPSS 25.0 (IBM Corp., Endicott, NY, USA) was used.

## 3. Results

A total of 71 patients were included during the recruitment period between September 2017 and January 2020. Three (4.2%) dropped out, so their data were removed, leaving 68 for the definitive analysis. The three dropouts were because these patients did not want to travel for a nutritional assessment (2) or blood test (1).

The analysis included 68 patients, 50 (73.35%) of whom were men, and the mean age was 64.25 ± 9.74 years. One-third of patients had type 2 diabetes (27.94%). Only nine patients were nonsmokers (13.24%). Most patients (72.1%; *n* = 49) had a high preoperative level of comorbidity, with a Charlson Index of 3 or more, implying a 3-year mortality of 52–58%.

The median preoperative FEV1 (maximal expiratory volume in the first second) was 100.44 ± 94.95 mL. Lobectomy was performed in 86.76% of the patients (*n* = 59). The most common histology was adenocarcinoma in 46 patients (63.24%), and most patients had pathological stage IB (*n* = 16; 23.52%). The remaining pathological, clinical, and surgical variables are reported in Table 1.

The main parameters of preoperative nutritional status and inflammatory status are presented in Table 2. The mean (±SD) preoperative patient weight was 75.61 ± 14.22 kg, and the mean preoperative BMI was 27.06 ± 4.96. Preoperative adherence to a Mediterranean diet was reported in 58.82% of the participants. The mean value of the primary study variable, omega 6/3 ratio, was 17.39 ± 9.45, and the mean vitamin D level was 35.78 ± 21.05 ng/mL. The median preoperative phA was 6.46 ± 2.1, and mean BCMI was 11 ± 2.33.

Postoperative complications occurred in 39.71% (*n* = 27) of the patients (Table 3), the most common being persistent air leak in 16 patients (23.53%), followed by respiratory failure (10.29%), surgical wound infection (7.35%), pneumonia (4.41%), and empyema (4.41%). All complications are reported in Table 3. None of the patients had deep vein thrombosis, pulmonary embolism, acute coronary syndrome, acute heart failure, stroke, vocal cord paralysis, bronchial fistula, or thoracic wall abscess, and none died during the study; there were no cases of postoperative hemothorax or chylothorax. The median hospital stay was 5.73 days (IQR = 4.68).

### Omega 6/3 Ratio

Analysis of the omega 6/3 ratio was performed in 59 patients (87%) due to losses in processing the samples. The median omega 6/3 ratio was 17.39 ± 9.45. The association between the omega 6/3 ratio and the rest of the variables was studied. For this, the variable omega 6/3 ratio was dichotomized, with a cutoff established at 21 based on a sensitivity study for air leak (AUC = 0.704; 95% CI: 0.53–0.84) that indicated this was the optimal cutoff, with a sensitivity of 83.8% and specificity of 70.2%.

**Figure 1:**
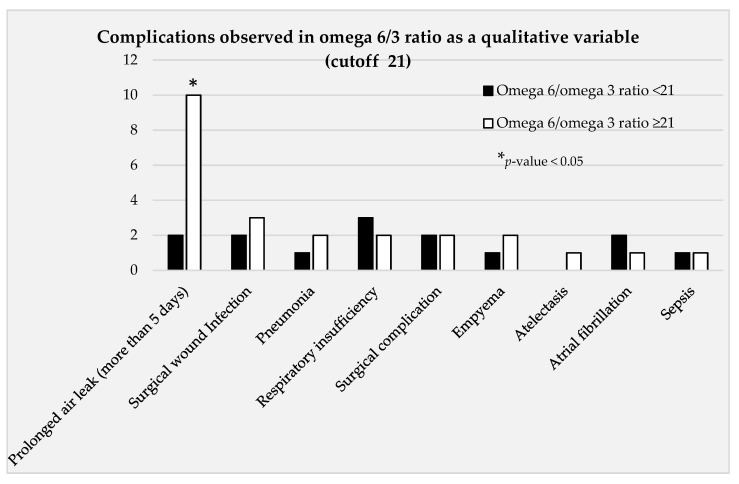
Bivariate analysis taking the variable omega 6/3 as a qualitative variable and study of complications.

A bivariate analysis was performed, analyzing the relationship between the omega 6/3 ratio as a qualitative variable (with a cutoff of 21) and the different complications observed (Figure 1*).* This bivariate analysis showed a statistically significant association between omega 6/3 ratio and persistent (≥5 days) air leak (*p* = 0.001) (Table 4).

Statistically significant differences (*p* = 0.004) were observed between the different scores on the Charlson comorbidity scale regarding the omega 6/3 ratio as a qualitative variable, the ratio being lower in those with less comorbidity (score 0–6) and higher in those with more comorbidity (score 7–8). There was also a statistically significant association with the variables sex, never smoker, COPD (chronic obstructive pulmonary disease), IPAQ at 1 year, and complications (Table 5).

We looked for differences in the association between omega 6/3 ratio and air leak according to the different surgical approaches used (thoracotomy vs. thoracoscopy) and found no significant differences. We also performed a breakdown of the different polyunsaturated fatty acids (eicosapentaenoic acid (EPA), docosahexaenoic acid (DHA), linolenic acid, arachidonic acid (ARA), and linoleic acid) and examined the association of these separately with the variable persistent air leak, finding no statistically significant results in the adjusted raw models.

In addition to its association with air leak, we found a statistically significant association between an omega 6/3 ratio of ≥21 and sex (*p* = 0.036), never smoker (*p* = 0.018), preoperative BCMI (*p* = 0.049), preoperative BMI (*p* = 0.015), IPAQ at 1 year (*p* = 0.047), hospital stay (*p* = 0.01), drain duration (*p* = 0.25), and overall postoperative complications (*p* = 0.006).

Before carrying out the multivariate analysis, a Bonferroni test was performed to identify potential interactions between the variables that had shown a statistically significant association with the omega 6/3 ratio in the bivariate analysis. This was conducted so that potential confounding variables could be considered when creating the models.

Subsequently, a multivariate model was built that included the variables sex, age, smoker, preoperative BCMI, preoperative BMI, drain duration, history of COPD, NPI, and persistent air leak postoperatively. This model explained the 79.2% between what was expected and what was observed. Of the variables that were significantly associated with the omega 6/3 ratio in the bivariate analysis, we excluded from the multivariate model: IPAQ at 1 year due to excessive loss of data and hospital length of stay, never smoker, and overall postoperative complications due to finding interactions. NPI was included in the model, even when it was not found to be significant in the bivariate analysis because it is an important variable for the thoracic surgery team. In the multivariate analysis using the omega 6/3 ratio with a cutoff of 21, the only variable with a continued significant association was COPD (*p* = 0.03; 95% CI: 1.22–53.57). The association with persistent air leak did not remain significant by a small margin (*p* = 0.052; 95% CI: 0.77–1.41).

The study of the relationship between preoperative markers of inflammation (such as NPI, SII, NLR, PLR, and LMR) and air leak found no statistically significant differences.

## 4. Discussion

The relationship between nutritional status and lung cancer has been the subject of research in recent years [11,12,13]. In the study carried out by Thomas et al. [11], they observed in a sample of 19,635 patients that those with a BMI of less than 18.5 had more pulmonary, surgical, and infectious complications. Among the surgical complications, air leak and dehiscence of the bronchial stump were the most common. In another study carried out in a population of over-70-year-old patients undergoing lung resection surgery for cancer, life expectancy was lower in those patients with a BMI less than 18.5, and the authors discussed in that article the importance of nutritional support in the pre- and postoperative periods [14]; such rehabilitation and nutritional support programs have been demonstrated to have benefits in terms of postoperative complications [15]. In a study performed by our team, we found that patients with low weight and/or malnutrition, as stratified by the nutritional risk index (NRI), had a longer hospital stay and that NRI was an independent predictor of postoperative complications [6]. In the present study, we also analyzed the relationship between NRI and postoperative complications and did not find a significant association, possibly due to the smaller number of patients included.

However, until now, only Kaya et al. [10] had assessed the specific role played by micronutrients, in particular omega essential fatty acids, in patients’ nutritional status and their relationship with postoperative outcomes in lung cancer. They described how a specific preoperative nutritional program with supplementation with omega-3 and other micronutrients led to improved postoperative recovery and a lower grade of postoperative complications. In this study, we observed a possible association between the preoperative omega 6/3 ratio and persistent air leak (≥5 days). Based on the results from this sample in the bivariate analysis, a lower preoperative omega 6/3 ratio may mean a lower probability of persistent air leak. Therefore, an increase in omega-3 (through diet or targeted supplementation) could have a protective function against air leak. The healing process is directly related to inflammation, an area in which the omega 6/3 ratio may exert an effect, immunomodulating the secretion of cytokines and altering their function on fibroblasts at a cellular level [16]. The relationships between the omega 6/3 ratio, air leak, and markers of systemic inflammation (CRP, LMR, platelets, NRI) were studied, but in this study, no conclusive results could be drawn. A parallel immunohistochemistry study is currently underway to study markers of local inflammation in the surgical tissue specimen in this same group of patients.

Multiple studies have described how the incidence and prevalence of cardiovascular disease, diabetes, asthma, and other diseases can vary depending on the proportion of omega-3 acids in the diet [8,9,17]. In the present study, we observed significant differences in the Charlson Comorbidity Index according to the omega 6/3 ratio, with a lower ratio in those with less comorbidity (score 0–6) and higher in those with more comorbidity, which could be explained by the relationship between this ratio and the prevalence of various diseases. However, few patients in this study had a high level of comorbidity.

Omega-3 fatty acids have also been demonstrated to reduce coronary disease, as they reduce levels of triglycerides and platelets [18]. The omega- 3 fatty acids EPA and DHA have been shown to inhibit tumor growth induced by TPA and epidermal growth factor (EGF) [19]. In lung cancer, the few studies that exist have focused on advanced stages, describing a positive effect in response to chemotherapy [20]. In the study by Cheng et al., they gave supplements with EPA and DHA or a placebo to 60 patients with lung cancer for 12 weeks and observed that the experimental group had a higher weight and reduction in inflammatory markers such as TNF and CRP compared with the control group [21]. None of these patients underwent surgery, which is where the importance of our study comes in, as it is the first to relate baseline omega fatty acid values to postoperative outcomes in lung cancer.

Persistent air leak is directly associated with a longer hospital stay, as can be observed from our results and as has been described in the literature [22,23]. This means an increased financial expenditure, which is why there is great interest in determining the factors that cause a greater air leak. These factors are divided into two groups, the first being the patient’s baseline characteristics, such as female sex, COPD, low FEV1, and history of smoking [24,25]. These variables were included in a multivariate analysis of the effect of the omega 6/3 ratio and persistent air leak, and a statistically significant association was observed. The second group is external factors, such as nutrition, as discussed above, and surgical technique. Benefits have been described with some maneuvers, such as fissureless surgery [26], staple length, and type of resection [25,26]. In our database, all patients were operated on according to the routine clinical practice of the surgeons at this hospital, with the same surgical technique and via a fissureless approach. Regarding the different approaches, there were no differences in the association between the omega ratio and persistent air leak according to the type of approach.

Despite these results, the need for supplementation with omega-3 in patients who are to undergo lung cancer surgery still requires further research, as there are some controversies: in the most recent meta-analysis by Lam et al. [27] on omega-3 supplementation in patients with cancer, they concluded that this supplementation did not improve patients’ quality of life, muscle mass, or weight, in contrast to what had been suggested in previous studies, although that analysis included all types of patients with cancer, while our group of patients had early-stage lung cancer and were candidates for radical surgical treatment.

It should be noted that the present study has some limitations, as it is an observational study and not a randomized controlled trial, as well as the obvious limitations due to the sample size; however, the findings from this sample show the importance of micronutrient analysis, particularly omega fatty acids. We recommend a preoperative assessment of omega fatty acid status with the goal of optimizing nutritional status, which could reduce the overall incidence of complications.

## 5. Conclusions

According to our findings, the omega 6/3 ratio in our population is high and could be correlated with immediate postsurgical complications.

## Figures and Tables

**Table 1:** Clinicopathological and surgical characteristics of the patients.

Variable	N (%)
Age	64.25 (±9.74) *
Male sex	50 (73.52%)
Active smoker	18 (26.47%)
Never smoker	9 (13.245)
Exsmoker	22 (32.35%)
Year packages	34.36 (±21.14)
Diabetes	19 (27.94%)
COPD	19 (27.94%)
Ischemic heart disease	6 (8.82%)
With previous pulmonary neoplasia	1 (1.47%)
Without previous pulmonary neoplasia	21 (30.88%)
Cerebral vascular accident (CVA)	3 (4.41%)
Peripheral vasculopathy	6 (8.82%)
Previous cardiac surgery	1 (1.47%)
Nephropathy	4 (5.88%)
Dyslipidemia	28 (41.18%)
Hypertension	35 (51.47%)
**Charlson Index**	
0	2 (2.94%)
1	6 (8.82%)
2	11 (16.18%)
3	13 (19.12%)
4	14 (29.59%)
5	12 (17.65%)
6 or more	10 (14.71%)
Thoracoscore (predicted mortality)	3.95 (±0.99)
Maximal expiratory volume in the first second (FEV1)	100.44 (±94.95) *
Histology	
Adenocarcinoma	43 (63.24%)
Squamous cell carcinoma	12 (17.65%)
Carcinoide tumor	2 (2.94%)
Large cell	4 (5.88%)
Benign	6 (8.82%)
Metastasis CCR	3 (4.41%)
**Pathological staging**	
Ia1	3 (4.41%)
Ia2	11(16.17%)
Ia3	6 (8.82%)
Ib	16 (23.52%)
IIa	3 (4.41%)
IIb	13 (19.12%)
IIIa	7 (10.29%)
IIIb	2 (2.94%)
Surgery	
Lobectomy	59 (86.76%)
Wedge	8 (11.76%)
Bilobectomy	1 (1.47%)
Approach	
Thoracotomy	35 (51.47%)
Thoracoscopy	31 (45.59%)
RATS (robotic-assisted thoracic surgery)	2 (2.94%)

* IQR, median ± interquartile range.

**Table 2:** Preoperative nutritional and inflammatory study.

Variable	Mean or Median (SD or IQR)
Height	1.66 (±0.09)
Weight	75.61 (±14.22)
PhA grades	6.46 (±2.10) *
BCMI (body cell mass index)	11 (±2.33)
BCM (body composition monitor) (kg)	30.29 (±8.01)
FFM (fat-free mass) (kg)	54.6 (±9.92)
FM (fat mass) (kg)	19.52 (±8.92)
MST (multistage testing)	12 (±17.65)
Adherence to the Mediterranean diet test	40 (±58.82)
BMI (body mass index)	27.06 (±4.96)
IPN (prognostic nutritional index)	47.1 (±5.85)
Preoperative Plasmatic Test	
Albumin	39.63 (±3.75) *
Prealbumin	225.05 (±67.69) *
Cholesterol	34.64 (117.91)
Vitamin D	35.78 (±21.05) *
Ácidos Grasos	
Ratio omega 6/3	17.39 (±9.45)
EPA	0.36 (±0.29)
DHA	1.23 (±0.71)
Linolenic acid	0.41 (±0.27)
ARA	6.33 (±1.90)
Linoleic acid	24.07 (±7.40)
Inflammatory Parameters	
Neutrophils	4720.44 (±2398.10)
Lymphocytes	1561.03 (±779.70)
Platelets	205,177.94 (±79,743.10)
Monocytes	525.59 (±206.60)
Ratio neutrophils/lymphocytes	3.91 (±3.64)
Ratio platelets/lymphocytes	152.32 (±70.36)
Ratio lymphocytes/monocytes	3.3 (±1.64)
Transferrin	41.66 (±58.30)
PCR	9.04 (±16.22)
Fibrinogen	4.05 (±51.63) *
SII (systemic immune-inflammation index)	12,162.21 (±581,087.45)

* IQR, median ± interquartile range.

**Table 3:** Postoperative complications.

Variable	N (%)
Any complication	27 (39.71%)
Atelectasis	2 (2.94%)
Pneumonia	3 (4.41%)
Respiratory insufficiency	7 (10.29%)
Atrial fibrillation	3 (4.41%)
Sepsis	2 (2.94%)
Fever (without pneumonia)	1 (1.47%)
Urinary infection	1 (1.47%)
Surgical complication	4 (5.885%)
Prolonged air leak (more than 5 days)	16 (23.54%)
Empyema	3 (4.41%)
Surgical wound infection	5 (7.35%)
Drainage days	4.70 (±8.16)
Length of stay	5.73 (±4.68) *
Recurrence	15 (21.15%)
Exitus	5 (7.4%)
**Clavien–Dindo Classification**	
None	41 (60.29%)
Grade I	23 (33.82%)
Grade II	2 (2.94%)
Grade IIIa	2 (2.94%)
Grade IIIb or higher	0

* IQR, median ± interquartile range.

**Table 4:** Bivariate analysis taking the variable omega 6/3 as a qualitative variable and study of complications.

Ratio Omega 6/3		<21 (*n* = 35)		≥21 (*n* = 24)		*p*-Value
		N	%	N	%
Complications	No	27	77.1	10	41.7	0.006
	Yes	8	22.9	14	58.3	
Atelectasis	No	35	100	23	95.8	0.223
	Yes	0	0	1	4.2	
Pneumonia	No	34	97.1	22	91.7	0.347
	Yes	1	2.9	2	8.3	
Respiratory insufficiency	No	32	91.4	22	91.7	0.974
	Yes	3	8.6	2	8.3	
Atrial fibrillation	No	33	94.3	23	95.8	0.790
	Yes	2	5.7	1	4.2	
Sepsis	No	34	97.1	23	95.8	0.785
	Yes	1	2.9	1	4.2	
Fever (without pneumonia)	No	34	97.1	24	100	0.404
	Yes	1	2.9	0	0	
Surgical complication	No	33	94.3	22	91.7	0.694
	Yes	2	5.7	2	8.3	
Prolonged air leak (more than 5 days)	No	33	94.3	14	58.3	0.001
	Yes	2	5.7	10	41.7	
Empyema	No	34	97.1	22	91.7	0.347
	Yes	1	2.9	2	8.3	
Surgical wound infection	No	33	94.3	21	87.5	0.358
	Yes	2	5.7	3	12.5	

**Table 5:** Bivariate analysis taking the variable omega 6/3 as a qualitative variable and baseline characteristics.

Ratio Omega 6/3		<21 (*n* = 35)	≥21 (*n* = 24)	
		Mean or Median (SD or IQR)	Mean or Median (SD or IQR)	*p*-Value
Age		65 (45)	64.5 (34)	0.877
Sex	Female	13 (37.1)	3 (12.5)	0.036
	Male	22 (62.9)	21 (87.5)	
Smoker	No	28 (80)	14 (58.3)	0.071
	Yes	7 (20)	10 (41.7)	
Never smoked	No	27 (77.1)	24 (100)	0.012
	Yes	8 (22.9)	0.0	
Exsmoker	No	28 (80)	16 (66.7)	0.248
	Yes	7 (20)	8 (33.3)	
Year packages		26 (21)	45 (26)	0.005
Diabetes	No	24 (68.6)	17 (70.8)	0.853
	Yes	11 (31.4)	7 (29.2)	
COPD	No	30 (85.7)	14 (58.3)	0.018
	Yes	5 (14.3)	10 (41.7)	
Ischemic heart disease	No	32 (91.4)	22 (91.7)	0.974
	Yes	3 (8.6)	2 (8.3)	
With previous pulmonary neoplasia	No	34 (97.1)	24 (100)	0.404
	Yes	1 (2.9)	0.0	
Without previous pulmonary neoplasia	No	22 (62.9)	17 (70.8)	0.525
	Yes	13 (37.1)	7 (29.2)	
Cerebral vascular accident (CVA)	No	34 (97.1)	22 (91.7)	0.347
	Yes	1 (2.9)	2 (8.3)	
Peripheral vasculopathy	No	33 (94.3)	22 (91.7)	0.694
	Yes	2 (5.7)	2 (8.3)	
Previous cardiac surgery	No	35 (100)	23 (95.8)	0.223
	Yes	0.0	1 (4.2)	
Nephropathy	No	33 (94.3)	23 (95.8)	0.79
	Yes	2 (5.7)	1 (4.2)	
Dyslipidemia	No	18 (51.4)	18 (75)	0.068
	Yes	17 (48.6)	6 (25)	
Hypertension	No	15 (42.9)	15 (62.5)	0.138
	Yes	20 (57.1)	9 (37.5)	
Charlson Index	0	1 (2.9)	1 (4.2)	0.266
	1	4 (11.4)	1 (4.2)	
	2	5 (14.3)	4 (16.7)	
	3	5 (14.3)	5 (20.8)	
	4	7 (20)	6 (25)	
	5	10 (28.6)	1 (4.2)	
	6	2 (5.7)	2 (8.3)	
	7	1 (2.9)	2 (8.3)	
	8	0.0	2 (8.3)	
VEMS		97 (501)	91.25 (629)	0.067

## Data Availability

The data presented in this study are available on request from the corresponding author. The data are not publicly available due to privacy reasons.
